# Level of daily physical activity in individuals with COPD compared with healthy controls

**DOI:** 10.1186/1465-9921-12-33

**Published:** 2011-03-22

**Authors:** Sigrid NW Vorrink, Helianthe SM Kort, Thierry Troosters, Jan-Willem J Lammers

**Affiliations:** 1 Utrecht University of Applied Sciences, Research Group Demand Driven Care, Bolognalaan 101, 3584 CJ Utrecht, The Netherlands; 2 Katholieke Universiteit Leuven, Research Centre for Cardiovascular and Respiratory Rehabilitation, Department of Rehabilitation Sciences, Herestraat 49, 00706 BE-3000 Leuven, Belgium; 3 University Medical Centre Utrecht, Division Heart and Lungs, Heidelberglaan 100, 3584 CX Utrecht, The Netherlands

## Abstract

**Background:**

Persons with Chronic Obstructive Pulmonary Disease (COPD), performing some level of regular physical activity, have a lower risk of both COPD-related hospital admissions and mortality. COPD patients of all stages seem to benefit from exercise training programs, thereby improving with respect to both exercise tolerance and symptoms of dyspnea and fatigue. Physical inactivity, which becomes more severe with increasing age, is a point of concern in healthy older adults. COPD might worsen this scenario, but it is unclear to what degree. This literature review aims to present the extent of the impact of COPD on objectively-measured daily physical activity (DPA). The focus is on the extent of the impact that COPD has on duration, intensity, and counts of DPA, as well as whether the severity of the disease has an additional influence on DPA.

**Results:**

A literature review was performed in the databases PubMed [MEDLINE], Picarta, PEDRO, ISI Web of Knowledge and Google scholar. After screening, 11 studies were identified as being relevant for comparison between COPD patients and healthy controls with respect to duration, intensity, and counts of DPA. Four more studies were found to be relevant to address the subject of the influence the severity of the disease may have on DPA. The average percentage of DPA of COPD patients vs. healthy control subjects for duration was 57%, for intensity 75%, and for activity counts 56%. Correlations of DPA and severity of the disease were low and/or not significant.

**Conclusions:**

From the results of this review, it appears that patients with COPD have a significantly reduced duration, intensity, and counts of DPA when compared to healthy control subjects. The intensity of DPA seems to be less affected by COPD than duration and counts. Judging from the results, it seems that severity of COPD is not strongly correlated with level of DPA. Future research should focus in more detail on the relation between COPD and duration, intensity, and counts of DPA, as well as the effect of disease severity on DPA, so that these relations become more understandable.

## Background

Chronic Obstructive Pulmonary Disease (COPD) is a disabling airway disease with variable extrapulmonary effects that may contribute to disease severity in individual patients. Its pulmonary component is characterized by airflow limitation that is not fully reversible. The airflow limitation is usually progressive and associated with an abnormal inflammatory response of the lung to noxious particles or gases. The characteristic symptoms of COPD are cough, sputum production, and dyspnea upon exertion [1].

Persons with COPD, who perform some level of regular physical activity, have a lower risk of both COPD-related hospital admissions and mortality [2,3]. COPD patients at all stages of the disease seem to benefit from exercise training programs, showing improvement with respect to both exercise tolerance and symptoms of dyspnea and fatigue [4]. Inactivity contributes to a further worsening of the physical condition of the subject and to even more dyspnea. This, in turn, contributes to a downward spiral of inactivity, deconditioning, and dyspnea [5,6]. COPD is a disease that mainly effects persons of older age. It develops over several decades of exposure to inhaled particulates, and, generally, becomes visible starting at the age of 40. In a global study, Buist et al. [7] found that the prevalence of COPD increases steadily with age for both men and women. The overall pooled Odds Ratio for COPD stage II or over was 1.94 (1.8-2.1) per 10-year age increment.

The American College of Sports Medicine and the American Heart Association provide physical activity recommendations for healthy older adults [8]. They recommend that each week older adults should do at least 30 minutes of moderate physical activity for 5 days or 20 minutes of vigorous physical activity for 3 days; 8-10 strength exercises for 2 days; and flexibility exercises for at least 10 minutes for 2 days. Many older adults do not meet these recommendations [9]. Caspersen et al. [10] studied the changes in physical activity patterns in the United States by sex and cross-sectional age. They found that both men and women reported more physical inactivity with greater age, with an increase between the 65-74-yr and >75-yr groups. After the age of 74, the prevalence of regular, sustained activity began to decline substantially for both sexes. Thus, physical inactivity, which becomes more serious with increasing age, is already a point of concern in healthy older adults. COPD might worsen this scenario, but it is unclear to what extent. There are various studies that examine the level of daily physical activities (DPA) in COPD patients. However, as of now, there is no overview of the literature that combines these studies to see to what extent the DPA of COPD patients differs from healthy individuals.

This literature review aims to present the extent of the impact of COPD on the DPA. The focus is on the extent of the impact that COPD has on duration, intensity, and counts of DPA, as well as whether the severity of the disease has an additional influence on DPA.

## Methods

Potentially relevant literature was identified through computerized searches. PubMed [MEDLINE], Picarta, PEDRO, ISI web of knowledge, and Google scholar were searched for articles. DPA was defined as: ''the totality of voluntary movement produced by skeletal muscles during every day functioning'' [5]. For this literature review we used the GOLD guidelines [1] to define disease severity. The severity of COPD is classified in four stages (mild to very severe) by postbronchodilator forced expiratory volume in 1 second (FEV1) value% predicted. The search was performed for the years 1966 to 2010 using the MeSH headings: "Pulmonary Disease, Chronic Obstructive", "Activities of Daily Living", "Motor Activity" and "Monitoring, Physiologic".

1. "Pulmonary Disease, Chronic Obstructive"[MeSH terms] AND "Activities of Daily Living"[MeSH terms] AND "Motor Activity"[MeSH terms] AND "Monitoring, Physiologic"[MeSH terms]/(8)

2. "Pulmonary Disease, Chronic Obstructive"[MeSH terms] AND "Activities of Daily Living"[MeSH terms] AND "Motor Activity"[MeSH terms]/(36)

3. "Pulmonary Disease, Chronic Obstructive"[MeSH terms] AND "Activities of Daily Living"[MeSH terms] AND "Monitoring, Physiologic"[MeSH terms]/(16)

4. "Pulmonary Disease, Chronic Obstructive"[MeSH terms] AND pedometer[All Fields]/(8)

5. "Pulmonary Disease, Chronic Obstructive"[MeSH terms] AND accelerometer[All Fields]/(20)

6. "Pulmonary Disease, Chronic Obstructive"[MeSH terms] AND activity[All Fields] AND monitors[All Fields])/(8)

7. "Pulmonary Disease, Chronic Obstructive"[MeSH terms] AND monitors[All Fields]/(14)

Inclusion criteria were as follows: 1) patients were diagnosed with COPD. 2) The study included a control group consisting of age-matched healthy subjects. 3) DPA was measured with an objective tool. 4) There was at least one whole day of measurements to be able to draw conclusions about *daily *physical activity.

Exclusion criteria were as follows: 1) DPA measured by questionnaires or diaries. 2) Measures on exercise capacity (for instance six minute walk test). These measures reflect one's *capacity *to perform physical activity in daily living, but give no information on the degree of DPA that is actually performed. 3) Measures on energy expenditure. Energy expenditure does not provide a direct measure of DPA. This is especially true in COPD patients because they exhibit an increased total daily energy expenditure while their daily physical activity level is reduced in comparison with healthy subjects [11].

Inclusion criterion 2 was not used in the search for articles on the effect of the severity of COPD on DPA. Articles were selected on the basis of title and abstract. References of obtained articles were verified whether they yielded any potentially relevant literature. After selection of the articles, full-text versions were obtained and read in their entirety after which, articles were either included or excluded based on the in- and exclusion criteria formulated above.

## Results

With the combinations of the keywords used, there were a number of 110 hits in total, 92 abstracts of which were screened. After screening, 11 studies were included based on the in- and exclusion criteria for comparison between COPD patients and healthy controls with respect to duration, intensity and counts of DPA. Two [12,13] were excluded because they measured DPA with questionnaires instead of objective measurements, resulting in 9 studies [14-22] used for data-extraction. Initially, the objective was to perform a meta-analysis with the data from all studies using the software package RevMan 5. Subgroup analyses with age, gender and disease severity were planned. Unfortunately, three studies were based on non-parametric data, and one study did not provide means and standard deviations [18]. This would leave 5 studies for analysis, which was decided to be too limited a basis to perform a meta-analysis. Instead, a descriptive review was written with 9 studies. In addition, four more studies [3,23-25] were included in this review as they address the subject of the influence the severity of the disease may have on DPA. These additional studies did not compare the COPD patients with healthy control subjects.

The total number of subjects in all studies was 766; 597 COPD patients and 169 healthy controls. Although different outcome measures were used in the various studies, they all represented an objective measure of DPA. The results were expressed in percentage of time of total recording time of DPA spent in various intensities [14,23], vector magnitude units (VMU) (sum of the three axes of the accelerometer) [16,24,25], walking time in minutes [15,17], mean number of movements per day [19], steps per day [3,21], mean activity count [18], total activity count [20], and mean activity (×10^3 ^counts/h) [22]. Study characteristics are shown in Table 1. This Table shows all 13 studies included for this review. The four studies analyzed with respect to disease severity display solely patient characteristics since these did not include healthy controls. Results of the individual studies are shown in Table 2.

**Table 1:** Study characteristics of included trials

Reference	N (P/C)	Mean Age (yrs±SD, P;C)	Gender (Male/Female) (P/C)	Variation in GOLD stage	Measurement device	Duration of study (days)
Coronado (2003)	25 (15/10)	67 ± 9; 57 ± 5	13:2; 4:6	1-4	Uni-axial accelerometer	1

Hernandes (2009)	70 (40/30)	66 ± 8;64 ± 7	18:22; 14:16	2-4	Tri-axial accelerometer	2

Lores (2006)	35 (23/12)	62 ± 7; 59 ± 7	20:3; 9:3	2-4	Tri-axial accelerometer	3

Pitta (2005)	75 (50/25)	64 ± 7; 66 ± 5	36:14; 17:8	1-4	Tri-axial accelerometer	2

Pitta (2008)	40	68 ± 7	21:19	1-4	Multisensor armband	2

Sandland (2005)	20 (10/10)	nd	nd	3	Uni-axial accelerometer	7

Schönhofer (1997)	50 (25/25)	56 ± 12; 53 ± 14	14:11; 14:11	2-3	Pedometer	7

Singh (2001)	20 (11/9)	66 ± 9; 59 ± 4	7:4; 5:4	3	Uni-axial accelerometer	2

Steele (2000)	47	66 ± 8	44:3	1-4	Tri-axial accelerometer	3

Steele (2003)	63	nd	nd	nd	Accelerometer	3

Troosters (2010)	100 (70/30)	66 ± 9; 65 ± 7	58:12; 19:11	1-4	Multisensor armband	6-8

Walker (2008)	51 (33/18)	67 ± 8; 70 ± 6	17:16; 8:10	3	Uni-axial accelerometer	3

Watz (2008)	170	64 ± 6.6	128:42	1-4	Multisensor armband	5-6

N = number of subjects, P = patients, C = controls, yrs = years, SD = standard deviation, nd = not described.

**Table 2:** Results of included studies

Reference	Duration of DPA			Intensity of DPA			Counts of DPA		
	
	P	C	P/C × 100%	P	C	P/C × 100%	P	C	P/C × 100%
Coronado (2003)	17% of recording time active	33%	52%						

Hernandes (2009)	55 ± 33 min/day walking	80 ± 28	69%	1.9 ± 0.4 m/s^2^	2.3 ± 0.6	83%			

Lores (2006)							184 ± 99 counts/3 days	314 ± 75	59%

Pitta (2005)	44 ± 26 min/day walking	81 ± 26	54%	1.8 ± 0.3 m/s^2^	2.4 ± 0.5	75%			

Schönhofer (1997)							3781 ± 2320 movements/day	8590 ± 4060	44%

Singh (2001)	136.1 min/2 days walking	386.2	35%				14.838 ± 7115 counts/2 days	24.028 ± 12399	62%

Troosters (2010)	106 min/day active	232	46%				5584 ± 3360 steps/day	9372 ± 3574	60%

Walker (2008)	50.8% ± 15.4% of recording time mobile	61.4% ± 11.2%	83%	156 ± 63 × 10^3 ^counts/h	232 ± 90	67%	82 ± 49 × 10^3 ^counts/h	143 ± 61	57%

Average			57%			75%			56%

DPA = daily physical activity, P = patients, C = controls, P/C × 100% = percentage DPA patients perform in comparison to healthy controls, value after ± is the standard deviation.

### The effects of COPD on duration, intensity, and counts of daily physical activity

To make a comparison between the studies with respect to the effect of COPD on duration, intensity and counts of DPA, the activity outcomes of COPD patients are given as a percentage of control values (Table 2). For example, in the study of Pitta et al. [17], the COPD patients walked for 44 minutes per day compared to 81 minutes of the healthy controls; 44 was then divided by 81 to come to a DPA percentage of 54 of COPD patients versus their healthy controls.

### Duration of DPA

Coronado et al. [14] showed in their study that the COPD patients were physically active for significantly less time of the day than healthy control subjects in low intensity activity (13% ± 4% vs. 22% ± 7%; p = 0.0001), and medium intensity activity (4% ± 4% vs. 11% ± 9%; p = 0.01). Hernandes et al. [15] evaluated the characteristics of physical activities in daily life in COPD patients in Brazil for 12 hours on two consecutive days. Mean walking time per day was shorter for COPD patients than for the controls (55 ± 33 vs. 80 ± 28 minutes per day; p = 0.001). In the study of Pitta et al. [17] patients also showed lower walking time than controls (44 ± 26 vs. 81 ± 26 minutes per day; p < 0.0001). Walker et al. [22] determined that patients spent significantly less time of the day being mobile than the controls (50.8% ± 15.4% vs. 61.4% ± 11.2% of recording time). Singh et al. [20] examined the discriminatory properties of an activity monitor by looking at the overall level of activity generated over two days by patients with COPD and healthy subjects. Patients completed a mean of 36.1 ± 17.1 minutes of brisk walking over the 48 hour period, whereas the healthy group completed 29.6 ± 41.4 minutes (p < 0.05). Patients, furthermore, performed 100 ± 64.3 minutes of slow or intermittent walking, whereas healthy subjects achieved a mean of 356.6 ± 219 minutes (p < 0.05). Troosters et al. [21] investigated DPA in patients with COPD and controls. Subjects were instructed to wear a multisensor armband device able to assess physical activity continuously (day and night) for six to eight days. The time spent in activities with mild (80 ± 69 minutes vs. 160 ± 89 minutes, p < 0.0001), moderate (24 ± 29 minutes vs. 65 ± 70 minutes; p < 0.0036), and high intensity (2 ± 5 minutes per day vs. 7 ± 9 minutes per day; P = 0.01) was significantly reduced in patients compared to controls. Table 2 shows the values of duration of DPA for the studies that include results on this parameter. Some studies reporting on the duration of DPA distinguish between various intensities at which activities were carried out. These are summarized in Table 2 to provide a single value of duration of DPA over a certain period of time. The ratio of duration of being active for healthy controls vs. patients with COPD is 1:0.57.

### Intensity of DPA

Hernandes et al. [26] showed that movement intensity was lower in patients vs. controls (1.9 ± 0.4 vs. 2.3 ± 0.6 m/s^2^; p = 0.004). In the study of Pitta et al. [17] patients showed lower movement intensity during walking (1.8 ± 0.3 vs. 2.4 ± 0.5 m/s^2^; p < 0.0001). Walker et al. [22] determined the relationship between lower limb activity and total DPA and related DPA to laboratory assessments before and after rehabilitation. The patients had a lower intensity of activity score (156 ± 63 vs. 232 ± 90 × 10^3 ^counts/h) than the healthy volunteers. The study of Coronado et al. [14] assessed the usefulness of an accelerometer to characterize walking activity during a 3-week rehabilitation program. They recorded patients as well as healthy subjects with an uni-axial accelerometer on the first and last days of a rehabilitation program. The results of the first day showed that the COPD patients studied showed significantly less time spent in low and medium intensity activity. The values for high-intensity activity were negligible in all subjects and thus not reported. Specific values for intensity of DPA were not mentioned, only the percentage of time spent in a certain intensity of DPA (see previous paragraph). The same representation of results on intensity is seen in Troosters et al. [21] and Singh et al. [20]. The first study shows similar results to Coronado et al. [14]. In the study of Singh et al. [20] patients spent more time on brisk walking, but substantially less on slow or intermittent walking (see previous paragraph). The ratio of intensity of DPA for healthy controls versus patients with COPD is 1:0.75 (Table 2).

### Activity counts

Lores et al. [16] assessed the agreement between different measurements of mean DPA. All subjects wore a tri-axial accelerometer for 8 days. Activity counts in COPD patients were significantly lower than those of controls (184 ± 99 vs. 314 ± 75; p < 0.01). The mean number of movements per day for patients and controls in the study of Schönhofer et al. [19] was 3781 ± 2320 and 8590 ± 4060, respectively (p < 0.0001). They concluded that COPD patients had DPA levels much lower than the average DPA level recorded in age- and gender-matched healthy individuals. Also, the range of DPA was much greater in healthy subjects than in COPD patients. Sandland et al. [18] explored the regular level DPA in patients with COPD and healthy subjects during a 7-day study with accelerometers. Their results showed that there was a significant difference (p < 0.001) in the level of DPA between healthy controls and COPD patients. Exact numbers, however, were not mentioned in the article. They do mention that the activity counts in COPD patients compared to those in the healthy group were reduced by 49%. The total activity count for COPD patients in the study of Singh et al. [20] was significantly lower than the total activity count in healthy subjects (14.838 ± 7115 vs. 24.028 ± 12399; p < 0.05). Troosters et al. [21] showed that patients took significantly less steps per day (5584 ± 3360) compared to controls (9372 ± 3574; p < 0.0001). Walker et al. [22] also reported that patients had a lower activity count compared to healthy volunteers (82 ± 49 vs. 143 ± 61; P = 0.001). The ratio of counts of DPA for healthy controls versus patients with COPD is 1:0.56 (Table 2).

### Disease severity and daily physical activity

The study of Pitta et al. [17] looked at the relation between disease severity and level of DPA in their patients. They found that there was no significant difference between walking time in patients with GOLD stages I and II when compared to patients with GOLD stage III (P = 0.10) and GOLD stage IV (P = 0.19). Standing time was significantly lower in patients with GOLD stages III and IV. They concluded that it appears that the correlation between disease severity and physical activity in daily life is not strong. A different study by Pitta et al. [23] measured DPA for two days using a multisensor armband device in 40 COPD patients. They correlated FEV1 with sedentary activities (r = -0.26), moderate activities (r = 0.29) and vigorous activities (r = 0.31; P = 0.05). Only the correlation with vigorous activities proved to be significant.

Hernandes et al. [15] found that walking time per day was not significantly correlated with FEV1% predicted (r = 0.17). The time spent standing per day correlated positively with FEV1% predicted (r = 0.41; p < 0.01).

Steele et al. [24] recorded walking and DPA in a sample of GOLD stage III COPD patients. They found that the accelerometers' output (vibrations in m/s^2^) correlated positively with FEV1% predicted (r = 0.62; p < 0.001). A different study by Steele et al. [25] measured 63 outpatients with COPD for three days and found a significant correlation between accelerometer measured daily activity and FEV1% predicted (r = 0.37; p < 0.01)

In the study of Schönhofer et al. [19] patients with stable non-hypercapnic COPD, patients with respiratory failure, and healthy subjects were measured. For the COPD patients the number of movements per day was positively correlated with the FEV1% predicted (r = 0.54, p = 0.006).

In the study of Singh et al. [20], total activity count was moderately correlated with FEV1% predicted when measured with an activity monitor (r = 0.41) but not statistically significant.

Walker et al. [22] found a correlation of r = 0.57 (P < 0.01) between mean activity of the legs and FEV1. Percentage of time spent mobile compared with FEV1 showed a correlation of r = 0.51 (p < 0.01).

In the study of Troosters et al. [21], the number of steps reached 87% ± 34%, 71% ± 32%, 49% ± 34% and 29% ± 20% of control values in GOLD-stages I to IV, respectively. The time spent in activities at moderate intensity was 53% ± 47%, 41% ± 45%, 31% ± 47% and 22% ± 34% of the values obtained in controls, respectively, with increasing GOLD-stage. These differences were statistical significant as of GOLD stage II (p < 0.05). The authors concluded that physical activity is reduced early in the disease progression (as of GOLD-stage II), and that reductions in physical activities at moderate intensity seem to precede the reduction in the amount of physical activities at lower intensity.

Watz et al. [3] measured DPA for five to six consecutive days using a multisensor armband device in 170 stable COPD outpatients. Steps per day diminished with increasing GOLD level (I:7990 ± 3370, II:7160 ± 3284, III:5126 ± 3692, IV:2377 ± 1897; p < 0.001). The same pattern was seen for mean physical activity level (I:1.63 ± 0.25, II:1.62 ± 0.27, III:1.45 ± 0.25, IV:1.27 ± 0.17; p < 0.001).

## Discussion

First, the reason for excluding the two studies that measured DPA with questionnaires will be elucidated. Studies that measured DPA by questionnaires or diaries were excluded because these measures represent subjective DPA. COPD patients significantly overestimate time spent walking and underestimate time spent standing [5,24,27], which makes results obtained via this method unreliable. The study of Gosker et al. [12] assessed DPA administering the Physical Activity Scale for Elderly (PASE) questionnaire. They found that the 25 COPD patients scored significantly lower (p < 0.001) than the 36 healthy gender- and age-matched controls; a lower score indicating a lower level of physical activity. Inal-Ince et al. [13] used the Activities of Daily Living Questionnaire (ADL-Q) in 30 COPD patients and 30 healthy controls. The ADL-Q scores were significantly higher in COPD patients (p < 0.0001), indicating a more pronounced inability to perform activities of daily living. The two excluded studies are in line with the results of this review, thereby lowering the chances of selection bias.

### The effect of COPD on daily physical activity

Table 2 shows similar results for all studies: COPD patients have lower levels of duration, intensity, and counts of DPA compared to healthy subjects. The average of the percentage of DPA of COPD patients vs. controls for duration was 57%, for intensity, 75%, and for activity counts, 56%. This shows that DPA is significantly affected by COPD. When converting the results of the studies to these percentages, it has to be taken into account that the values used in this calculation are mean values with large standard deviations for the COPD patients as well as for the healthy controls. It seems that the large differences between persons in level of DPA is not caused by the disease but merely by individual differences.

Next to a shorter duration of activity, the included studies also reported longer durations of inactivity. The COPD patients tended to spend more time seated than controls (294 ± 114 vs. 246 ± 122 min/day, p = 0.08) in Hernandes' study [15] as well as in Pitta's (374 ± 139 vs. 306 ± 108 minutes/day; p = 0.04) [17]. This latter study also reported a higher lying time (87 ± 97 vs. 29 ± 33 min/day; p = 0.004). Furthermore, they reported a lower standing time (191 ± 99 vs. 295 ± 109 min/day; p < 0.0001). The study of Coronado et al. [14] showed that the COPD patients studied were significantly physically inactive for longer periods of time than the healthy control subjects (82% of recording time vs. 68% for controls).

It might be expected that having COPD would cause one to achieve the same duration of DPA, albeit at a lower intensity. Interestingly, intensity of DPA seems to be less affected by COPD than duration and counts. This trend is also evident in the study of Singh et al. [20]. The patients performed less physical activity overall, but interestingly enough, they completed more minutes of brisk walking than the healthy controls. It could be that the patients are trying to perform activities as fast as possible so as to alleviate the unease caused by physical activity. This puts a lot of strain on their bodies, which are already weaker as a result of the disease. The longer duration of inactivity seen in the studies of Hernandes et al. [15] and Pitta et al. [17] could be a result of this behavior. If trained to perform activities at a lower intensity, they might be able to improve their overall duration and count of DPA.

### Disease severity and daily physical activity

Overall there seems to be a relation between DPA and severity of the disease, but this correlation is moderate at most and certainly not strong. Aliverti and Macklem [28] mention that shortness of breath is not necessarily the primary factor that limits exercise. They hypothesize that that both respiratory muscles and skeletal muscles do not receive sufficient oxygen to continue metabolizing aerobically. The fact that some individuals terminate exercising because of dyspnea, whereas others stop because of leg fatigue, suggests that sometimes the legs receive more oxygen and other times, the respiratory muscles. This would explain this moderate correlation between the severity of COPD based on the GOLD classification and DPA.

There has been some discussion about whether FEV1% predicted is a good variable to classify disease severity in COPD. It does not correlate well with important outcomes such as symptoms, quality of life, survival, exacerbation frequency, and exercise tolerance [29-31]. A different classification model might better correlate disease severity with DPA. In the study of Sandland et al. [18], there was a significant reduction in the level of DPA in the group of COPD patients with long term oxygen therapy (LTOT) compared with the group without LTOT. The two groups had the same level of disease severity according to the GOLD staging system. The difference between the two groups was the LTOT, which indicates that the group with LTOT had more severe hypoxemia and thus more symptoms. Here, the increase in severity of symptoms independent of disease severity according to the GOLD classification seems to further worsen the level of DPA. It seems important that when prescribing exercise training to a COPD patient, one takes into account that a more severely inflicted patient does not necessarily need a less physically demanding program. The patient might be able to perform on a higher level than one might think judging by their disease severity measured by FEV1 (% predicted).

### Limitations

The selection of patients and controls varied between the studies, which may constitute an important source of bias. Patients who were recruited were either participating in an inpatient rehabilitation program [14,15,18,22], an outpatient rehabilitation program [16,19,21], just graduated from a rehabilitation program [20], or recruitment of patients was not clearly stated [17]. Patients referred to rehabilitation programs may be more likely to be inactive than the general population of COPD patients. This latter group may prevent themselves from entering a period of rehabilitation because they are more physically active and thereby maintain a better health status. The selection of the healthy controls was not always clearly described. Coronado et al. [14] included subjects, who participated on a voluntary basis from a senior group practicing fitness. Pitta et al. [17] recruited relatives and friends from the researchers and patients. Hernandes et al. [15] and Troosters et al. [21] recruited relatives of students of the university. A random sample of healthy persons may be more inactive than volunteers, which would diminish the difference between COPD patients and their healthy controls.

It was decided that too few studies were available in order to perform a meta-analysis. This is due to limited research that is specifically directed at the relation between COPD and duration, intensity, and counts of DPA, as well as the effect of disease severity on DPA. Future research should focus in more detail on this subject so that these relations become more understandable.

Apart from the fact that there were too few studies for a meta-analysis, another difficulty in the comparison of the studies was that there was a great variety in the instruments used to obtain an objective measure of daily level of physical activity. When comparing studies with different kinds of pedometers and accelerometers, this can lead to some variation in the outcome that is not attributable to the subjects but rather to the different measurement devices. The methods of the studies also differed considerably in measurement time. However, since the difference in daily physical activity between COPD patients and healthy controls was so large in the included studies, the effect of various methodologies is probably negligible.

## Conclusion

Even though there was a great variation between the studies in terms of measurement devices and measurement time, the large differences between the healthy subject group and the COPD patient group allow us to draw conclusions. From the results of this review, it appears that patients with COPD have a significantly reduced duration, intensity, and counts of DPA when compared with healthy control subjects. Intensity of DPA seems less affected by COPD than duration and counts. Judging from the results, it seems that severity of COPD is not strongly correlated with level of DPA. Future research should focus in more detail on the relation between COPD and duration, intensity, and counts of DPA, as well as the effect of disease severity on DPA, so that these relations become more understandable.

## Abbreviations

COPD: Chronic Obstructive Pulmonary Disease; DPA: Daily Physical Activity; GOLD: the Global Initiative for Chronic Obstructive Lung Disease; FEV1: Forced expiratory volume in one second; LTOT: Long Term Oxygen Therapy; MVV: Maximum Voluntary Volume; IC: Inspiratory Capacity; FVC: Forced Vital Capacity.

## Competing interests

The authors declare that they have no competing interests.

## Authors' contributions

SV conceived of the study, drafted the manuscript. HK conceived of the study and helped to draft the manuscript. TT conceived of the study and helped to draft the manuscript. JL conceived of the study and helped to draft the manuscript. All authors read and approved the final manuscript.
